# Application of PCR-Based Techniques for the Identification of Genetic Fingerprint Diversity of Dominant Bacteria in Fecal Samples of Children with Diarrhea in Vietnam

**DOI:** 10.3390/idr16050075

**Published:** 2024-09-29

**Authors:** Thi Quy Nguyen, Trong Khoa Dao, Hong Duong Nguyen, Thi Bich Thuy Phung, Thi Thanh Nga Pham, Thi Viet Ha Nguyen, Thi Huong Trinh, Huu Cuong Le, Thi Thu Hong Le, Thi Huyen Do

**Affiliations:** 1 Institute of Biotechnology, Vietnam Academy of Science and Technology (VAST), 18-Hoang Quoc Viet, Cau Giay, Ha Noi 10072, Vietnam; quynhungcuong@yahoo.com (T.Q.N.); khoadt@ibt.ac.vn (T.K.D.); duongnguyen96uet@gmail.com (H.D.N.); lethuhong@ibt.ac.vn (T.T.H.L.); 2 Department of Molecular Biology for Infectious Disease, Vietnam National Children’s Hospital, 18/879 La Thanh, Dong Da, Ha Noi 11500, Vietnam; thuyphung.nhp@gmail.com (T.B.T.P.); huongtrinh206@gmail.com (T.H.T.); 3 Department of Gastroenterology, Vietnam National Children’s Hospital, 18/879 La Thanh, Dong Da, Ha Noi 11500, Vietnam; phamthithanhnga1986@gmail.com (T.T.N.P.); vietha@hmu.edu.vn (T.V.H.N.); 4 Department of Pediatrics, Hanoi Medical University, 1-Ton That Tung, Dong Da, Ha Noi 11500, Vietnam; 5 Institute of Natural Products Chemistry, Vietnam Academy of Science and Technology (VAST), 18-Hoang Quoc Viet, Cau Giay, Ha Noi 10072, Vietnam; lehcuong@inpc.vast.vn; 6 Graduate University of Science and Technology, Vietnam Academy of Science and Technology (VAST), 18-Hoang Quoc Viet, Cau Giay, Ha Noi 10072, Vietnam

**Keywords:** PCR-DGGE, bacterial dysbiosis, bacterial extraction, diarrhea, children, diversity indices, molecular fingerprinting techniques

## Abstract

In Vietnam, diarrhea, especially persistent diarrhea, is one of the most common diseases in children, while a significant proportion of cases are negative with pathogens; thus, there is an urgent need to understand gut bacterial dysbiosis. In this study, bacteria in the fecal samples of five healthy and ten diarrheal children were separated from other residues, then adopted to extract their metagenomic DNA for evaluating their diversity based on V3 and V6–V8 regions and the 16S rRNA gene by PCR-RFLP and PCR-DGGE. As a result, bacterial metagenomic DNAs with high quality, quantity and diversity were successfully extracted using a GeneJET kit and a chemical protocol. A sequence analysis of 73 representative DNA fragments from gels indicated a remarkable bacterial dysbiosis in all groups of diarrhea. Viral diarrhea was characterized by extremely reduced bacterial diversity with the blossom of *Bifidobacterium* and *Streptococcus*. *Streptococcus* was also the most abundant in persistent diarrhea. Beneficial bacteria that may play a role in the self- rebalance in intestinal bacterial communities, such as *Bifidobacterium*, *Lactobacillus,* and *Enterococcus,* were seen in all diarrheal groups, while *Bacteroides* and *Akkermansia muciniphila* were seen in the healthy group but absent in the diarrheal groups. This study provides additional evidence for a relationship between intestinal bacterial dysbiosis and diarrhea in children, emphasizing an increase in *Streptococcus*.

## 1. Introduction

Diarrhea is the third leading cause of death in children under 5 years old worldwide, especially in developing countries. Annually, the disease causes the death of 443,832 children among 1.7 billion cases [1]. Diarrheal disease is defined by loose and watery stools three or more times in 24 h. Acute diarrhea is the most common, lasting less than 2 weeks, while persistent diarrhea lasts between 2 and 4 weeks. In Vietnam, diarrhea is one of the most common diseases in children and can lead to mortality [2,3]. The main causes of diarrhea in infants are bacterial, viral and parasite infections. The pathogens can be detected by microbiological, immunological and molecular technologies. Multiplex real-time PCR is now adopted in Vietnam National Children’s Hospital to detect up to 24 pathogenic agents in fecal samples within 24 h. However, there is a significant proportion of diarrhea with unidentified pathogens. In 2016, no common pathogens could be determined in relation to about 47.39% of children suffering from persistent diarrhea [4]. The proportion of children with prolonged diarrhea leading to malnutrition was about 31%, of which 26.8% were underweight and 12.7% were malnourished and wasting [5]. The persistent diarrheal state may be promoted by malnutrition and other factors (especially antibiotic exposure), predisposing the sufferer to intestinal dysbiosis, leading to an increase in the selected populations of certain bacteria [6], impacts on intestinal mucosal structural integrity and immunity and on metabolic homeostasis [7]. Thereby, rather than infection, it is commensal gut microbiota dysbiosis that is suspected as an origin of persistent diarrhea. Previous studies have indicated an impressive transition of the bacterial community in the fecal samples of the following: (1) acute diarrheal pediatric patients with rotavirus infection; (2) patients with a mixed infection of rotavirus and *E. coli*; (3) patients with *E. coli* infection; (4) patients with an unidentified pathogen; and (5) patients with other infections. The authors emphasized that there was significant dysbiosis of the *Streptococus* population in each of the diarrheal groups [6,8]. This raises doubt about the pathogenic role of this genus. Thus, understanding the variation in bacterial communities in the gastrointestinal gut of diarrheal infant patients is of critical importance when seeking an appropriate strategy (treatment or complementary therapy) for individual children recovering from persistent diarrhea.

The development of culture-independent methods, such as next-generation sequencing (NGS) and molecular fingerprinting techniques, has made the characterization of microbial communities increasingly feasible and affordable. The NGS is the method of choice for the characterization of a microbial community composition based on the 16S rRNA gene [9]; however, this method is applied to separated samples and requires time-consuming analysis when comparing the variation in bacterial communities among many samples. Fingerprinting techniques, such as denaturing gradient gel electrophoresis (DGGE), restriction fragment-length polymorphism (RFLP), or single-strand conformation polymorphism (SSCP), can help to quickly understand, simultaneously and reproducibly with a large number of samples (for comparison and identification of variable trend response to any factor), the spatial and temporal variability in gastrointestinal microbial community structures [10]. The RFLP method is easy to perform and has high resolution but is suitable for analyzing bacterial populations at a low diversity [11,12,13]. The DGGE separates similar-sized DNA fragments based on their DNA melting property, which has been frequently reported for the identification of single-nucleotide polymorphisms without the requirement of DNA sequencing, thus being appropriate for complex ecosystem communities [14]. However, for the successful analysis of bacterial communities by molecular technology, the quality and quantity of metagenomic DNA extracted from bacteria in fecal samples play a pivotal role because fecal samples are commonly contaminated with various PCR inhibitors [15]. It has been proven that PCR inhibitors accumulate with age in a range that begins with 0% in the stool samples of newborns to 17% in the stool samples of children who are 6 to 24 months of age [16], with that proportion increasing in the samples of adults [17]. There are several kinds of PCR inhibitor, including phenolic compounds, such as antibiotics, fats, cellulose, constituents of bacterial degraded cells, heavy metals, etc. [16]; thus, the stool samples from diarrheal children might contain more PCR inhibitors than the samples from healthy children. Although there are many methods that have been reported to limit the impact of PCR inhibitors, such as DNA template dilution, adding chemicals in the PCR reaction, or improvements in the PCR program [17], we consider the removal of PCR inhibitors from bacteria to be important when seeking to obtain high-quality metagenomic DNA. Thus, in this study, we first separated bacteria from other fecal residues and then used the bacteria for the extraction of their metagenomic DNA.

To harvest a high quality and quantity of metagenomic DNA from stools, several procedures for the extraction of DNA have been described. Typically, a previous study adopted 21 commercial kits and found that QIAamp Stool Mini kit (Qiagen, Hilden, Germany) generated the best quality and quantity of metagenomic DNA extracted from fecal samples. However, the authors wondered about the applicability of this test for low-biomass fecal samples from newborn [18]. In addition, a higher quantity of metagenomic DNA could also be obtained when applying a biochemical protocol; however, the authors met problems with inhibitor contamination in the DNA [17]. In our previous study, we succeeded in the extraction of bacterial metagenomic DNA from a fecal sample of a child with acute diarrhea presenting symptoms of intestinal infection via a chemical method, as described by Sambrook [19,20]. In general, stool samples are very complex, contain many residues and PCR inhibitors and are greatly affected by diet, infectious status or/and medical treatment. Thus, in this study, we rechecked five methods for extracting the metagenomic DNA of fecal samples from children suffering from persistent diarrhea, then applied the most effective methods for extracting metagenomic DNAs from fecal samples of children with viral diarrhea, undetected pathogenic acute diarrhea and persistent diarrhea.

With regard to diarrhea in the absence of detected pathogens, the pathogenesis of the diarrhea remains poorly understood. Thus, in this report, after analysis of *Mbo*I-restricted 16S rRNA fragments in agarose gel (in the case of RFLP analysis) and V3, the V6–V8 regions of 16S rRNA gene in polyacrylamide gel (in the case of DGGE analysis), the typical DNA bands of every sample were harvested and sequenced to identify the abundant genus in the samples. The dysbiosis of fecal dominant bacteria in the group of diarrhea was analyzed.

## 2. Materials and Methods

### 2.1. Ethical Committee Approval

The study protocol was approved by the Ethics Committee of Vietnam National Children’s Hospital, Vietnam (No: 1875/BVNTW-HĐĐĐ signed on 10 July 2023). The children’s parents or caregivers were informed about the aim of the study and then signed a written informed consent.

### 2.2. Sample Collection

A total of 15 children under five years old, including 5 healthy and 10 diarrheal individuals, were enrolled in this research. The control children (control group, designated from C1 to C5) were healthy (not suffering from chronic diseases; had not manifested from infection, digestive disorder, diarrhea, or diseases related to the digestive tract; had not used antibiotics at least one month before sampling) and negative for the 24 common diarrheal pathogens by QIAstat-Dx Gastrointestinal Panel test that is described in detail below. The diarrheal groups were composed of acute, viral and persistent states, designated from D1 to D10, as listed in Table 1. Fecal samples (one from each subject) were collected based on the instruction of a handbook for use of testing services (ST.XN.4.4) No. 13/CT-BVNTW signed on 22 March 2023 of the Vietnam National Children’s Hospital. The feces without urine were transferred into plastic bottles and directly transported to the Department of Molecular Biology for Infectious Diseases, Vietnam National Children’s Hospital, to detect potential diarrheal pathogens via real-time PCR using QIAstat-Dx Gastrointestinal Panel (Qiagen, Hilden, Germany), Allplex™ GI-Bacteria(I) Assay (Seegene Inc., Seoul, Republic of Korea), Allplex™ GI-Bacteria(II) Assay (Seegene Inc., Seoul, Republic of Korea) and Allplex™ GI-Virus Assay (Seegene Inc., Seoul, Republic of Korea) within 24 h (Table 1). The QIAstat-Dx Gastrointestinal Panel (Qiagen, Hilden, Germany) can detect 24 common pathogens, including 4 parasites (*Entamoeba histolytica*, *Cryptosporidium* spp., *Giardia lamblia*, *Cyclospora cayetanensis*), 14 bacteria (*Vibrio vulnificus*, *V. parahaemolyticus*, *V. cholerae*, *Campylobacter* spp., *Salmonella* spp., *Clostridium difficile* (*tcdA*/*tcdB*), *Yersinia enterocolitica*, Enteroaggregative *E. coli* (EAEC), Enterotoxigenic *E. coli* (ETEC), Shiga-like toxin-producing *E. coli* (STEC), Shiga toxin-producing *E. coli* (STEC) serotype O157:H7, Enteropathogenic *E. coli* (EPEC), Enteroinvasive *E. coli* (EIEC)/*Shigella*, *Plesiomonas shigelloides*), and 6 viruses (human Adenovirus F40/F41, Norovirus GI, GII, Rotavirus A, Astrovirus, Sapovirus GI, GII, GIV, GV). The Allplex™ GI-Bacteria(I) Assay (Seegene Inc., Seoul, Republic of Korea) can diagnose seven bacteria, including *Aeromonas* spp., *Campylobacter* spp., *C. difficile* toxin B, *Salmonella* spp., *Shigella* spp./EIEC, *Vibrio* spp., *Yersinia enterocolitica*. GI-Bacteria(II) Assay (Seegene Inc., Seoul, Republic of Korea) can detect six bacteria, including EAEC, EPEC, *Escherichia coli* O157, ETEC, hypervirulent *Clostridium difficile*, STEC. Allplex™ GI-Virus Assay (Seegene Inc., Seoul, Republic of Korea) detects six viruses, including Astrovirus, Sapovirus, Rotavirus A, Norovirus GI, Norovirus GII, Adenovirus F. The remaining feces were kept at −80 °C. The samples included in this research were negative for common diarrheal bacterial pathogens based on the corresponding detection kits.

### 2.3. Harvest of Bacteria from Fecal Samples

In general, stool samples usually contain PCR inhibitors that may lead to false-negative results. In this study, 12 of the 15 samples were negative for diarrheal bacterial pathogens using the real-time PCR tests (Table 1); the total nucleic acid of the samples was automatically extracted by nucleic acid extraction kits [21], accompanied by the real-time PCR QIAstat-Dx system (Qiagen, Hilden, Germany). The extraction kit can eliminate part of the PCR inhibitors. Thus, to enhance the discarding of PCR inhibitors, first, we extracted bacterial fraction from the rest of the fecal samples; then, the bacteria were used for extraction of their metagenomic DNA using five methods.

The stool samples (1 g) from −80 °C were thawed on ice then mixed with 3 mL of cold PBS buffer (137 mM NaCl, 2.7 mM KCl, 10 mM Na_2_HPO_4_, 2 mM KH_2_PO_4_, pH 7.4), thoroughly homogenized via vortex, then kept on ice for 10 min to settle. The supernatants were carefully transferred into new falcon tubes. The remaining part in each tube was added with 3 mL of cold PBS buffer, followed by mixing thoroughly by vortex machine and centrifuged at 500 rpm for 10 min. The supernatants were correspondingly pooled with the supernatants in the previously collected falcon tubes. The samples were subjected to centrifuge at speeds from low to high, the first at 700 rpm for 10 min (replicated twice), followed by 1000 rpm to separate the bacterial phase in supernatants from the fecal residues (replicated three times). After centrifuging at 10,000 rpm for 5 min, the pellets of bacteria were washed with PBS and re-collected at the same centrifugation speed. The resulting bacterial pellets were well suspended in TE buffer (10 mM Tris, 1 mM EDTA, pH 8.0), and we measured the cell density at 600 nm with a NanoDrop NC-2000C Implen (Isogen, PW De Meern, The Netherlands), then divided 4 × 10^9^ CFU into each Eppendorf tube based on OD_600_ value, while OD_600_ = 1 was estimated to be equal to 1.5 × 10^9^ CFU/mL [22]. Finally, the cells were harvested by centrifugating at 10,000 rpm for 5 min and then stored at −20 °C for further DNA extraction.

### 2.4. Extraction of Bacterial Metagenomic DNA

#### 2.4.1. Investigation of Methods for Obtaining High Yield of Metagenomic DNA

In our previous study, we investigated five methods for extraction of bacterial metagenomic DNAs from fecal samples of a healthy boy (12 months old) and a diarrheal girl (15 months old) diagnosed with intestinal infection of unidentified cause [19]. Nevertheless, the fecal samples are complex, depending on nutrition and infected diseases; thus, the yield and quality of metagenomic DNA from different stools fluctuate [18]. Therefore, in this study, we rechecked methods for extraction of metagenomic DNA of bacteria in fecal samples C1 and D9, which were negative for 24 common diarrheal pathogens via QIAstat-Dx^®^ Gastrointestinal Panel (Qiagen, Hilden, Germany) (Table 1). Sample D9 was collected from a child who suffered from persistent diarrhea.

The bacterial samples (4 × 10^9^ cells) from −20 °C were taken, kept on ice for 15 min, then subjected to metagenomic DNA extraction using four kits, including GeneJET Genomic DNA purification kit (Thermo Scientific, Waltham, MA, USA), QIAamp Fast DNA Stool Mini Kit (Qiagen, Hilden, Germany), E.Z.N.A Stool DNA kit (Omega Bio-tek, Norcross, GA, USA), TopPURE stool DNA extraction kit (ABT, Ha Noi, Vietnam), in accordance with the manufacturer’s instructions. In the final step, metagenomic DNAs were eluted in 50 µL of the respective elution buffers. On the other hand, the samples were subjected to metagenomic DNA extraction by phenol/chloroform method (the chemical method), as described by Sambrook [19,20]. Finally, DNA pellets were reconstituted in 50 µL of TE buffer then checked by electrophoresis on agarose gel (0.8%). The quality and quantity of the DNAs were determined by a NanoDrop NC-2000C Implen (Isogen, PW De Meern, The Netherlands).

#### 2.4.2. Detection of Polymerase Inhibition by PCR

To check the presence of DNA polymerase inhibitors in the extracted metagenomic DNAs, the DNAs were used as templates for amplification of a gene coding for 16S rRNA (designated as 16S rRNA gene) using primers: 27F (5′-GAGTTTGATCCTGGCTCAG-3′) and 1527R (5′-AGAAAGGAGGTGATCCAGCC-3′). PCR reaction mixture (25 μL) contained 2.5 μL10× PCR buffer, 2 μL dNTP 2 mM, primers (0.4 μM each), 1.3 U of Taq DNA polymerase (Thermo Scientific, Waltham, MA, USA), dH_2_O, and DNA templates (either approximately 1 ng or 7 ng, 50 ng or 100 ng per reaction mixture). The amplification was conducted at 94 °C for 4 min, followed by 25 cycles of the template denaturation at 94 °C for 30 s, primer annealing at 55 °C for 30 s, and extension at 72 °C for 1 min. A final extension step was carried out at 72 °C for 4 min. The PCR products were analyzed by electrophoresis on agarose gel (0.8%).

#### 2.4.3. Selection of Methods for Extracting Diverse Metagenomic DNA from Stools

Because the metagenomic DNAs will be used for the assessment of bacterial diversity, we were interested in not only yield and quality but also the diversity of the metagenomic DNAs. To evaluate the diversity of the metagenomic DNAs extracted by different methods, PCR–RFLP was adopted. The metagene 16S rRNA was amplified by the same components as described above from 2 ng metagenomic DNAs extracted using five methods. The program for PCR was as follows: 94 °C for 4 min; five cycles of 94 °C for 30 s, 50 °C for 30 s, 72 °C for 1 min; five cycles of 94 °C for 30 s, 52 °C for 30 s, 72 °C for 1 min; five cycles of 94 °C for 30 s, 55 °C for 30 s, 72 °C for 1 min; 20 cycles of 94 °C for 30 s, 58 °C for 30 s, 72 °C for 1 min; and the final extension at 72 °C for 4 min. The PCR products were analyzed by electrophoresis on the agarose gel 0.8%, then digested by *Mbo*I. Total reaction (7.5 μL) comprised 0.75 μL buffer CutSmart 10×, 500 ng PCR products, 3 U *Mbo*I (New England Biolabs, Ipswich, MA, USA). The reaction was conducted at 37 °C for 3 h. The digested products were electrophoresed on 2.5% agarose gel, stained with ethidium bromide (0.05%), then visualized under UV light.

For analysis of bacterial diversity in the extracted metagenomic DNA samples, the electrophoresis picture was scanned by InfanView and analyzed by ImageLab to obtain densitometric curves, DNA bands and similarity indices. Levels of similarity of fingerprinting profiles among samples were calculated according to the Dice coefficient. The unweighted pair group method with arithmetic averages (UPGMA) was used to transform similarity coefficients into distance, subsequently creating a heatmap dendrogram [23]. The best extraction methods that gave the most diverse 16S rRNA result were selected for extraction of metagenomic DNAs from the 15 bacterial samples. The metagenomic DNAs were analyzed by electrophoresis on 0.8% agarose gel. The quality and quantity of the DNA were determined as indicated earlier.

### 2.5. Analysis of Bacterial Diversity in the Stool Samples Using Fingerprinting Techniques

Three fingerprinting techniques, including PCR-RFLP based on 16S rRNA metagene, PCR-DGGE based on the V3, V6–V8 regions of 16S rRNA metagene, were used for the assessment of bacterial diversity in the 15 stool samples. The PCR-RFLP was conducted as described above.

The DGGE was performed as described by Hovda et al. [24] with the VS20-DGGETC system (Cleaver Scientific Ltd., Warwickshire, UK). Firstly, the V3 (180 bp), V6–V8 (433 bp) were amplified by specific primers that were demonstrated in previous studies [25,26] using the PCR program. The PCR products (1 μg/sample) were loaded on a 0.75 mm thick 8% (*w*/*v*) polyacrylamide gel, with a denaturing gradient ranging from 35% to 70% for V3, from 40 to 60% for V6–V8. The electrophoresis was run in TAE buffer (Eppendorf AG, Hamburg, Germany) at 60 °C for 10 min at 20 V and for a further 18 h at 70 V. After electrophoresis, the gels were stained with SYBR Gold (S11494, Thermo Scientific, Waltham, MA, USA), diluted 10,000 times in TAE buffer pH 7.6. The gels were rinsed with water, and the DNA bands were visualized under UV light. The electrophoresis pictures were scanned for analysis of the bacterial diversity in the 15 samples, as described above. Diversity indices, including Shannon–Wiener diversity (H0), abundance or evenness (E) and Simpson’s diversity (D), of bacterial community structures in stool samples were assessed through the corresponding DGGE or RFLP banding patterns, as described by Sun et al. (2008) [27].

### 2.6. Sequencing Representative DNA Bands and Analysis of Bacterial Diversity

The typical DNA bands in each sample that were identified from the gels were excised and transferred to Eppendorf tubes containing 30 μL water, allowing DNA to diffuse into the water at 4°C for 1 h and then kept overnight at −80 °C. The samples were quickly thawed then centrifuged at mild speed (500 rpm–1000 rpm) for 2–5 min. The bands restricted by *Mbo*I from RFLP gel were cloned into pBluescript KS(-) digested with *Bam*HI and sequenced in the ABI 3100 by Sanger protocol using the universal primers M13 forward and M13 reverse. Meanwhile, the DNA fragments from DGGE gels were diluted maximally (10^−4^ to 10^−6^ folds by TE buffer) to be used as templates for PCR to amplify respective V3 or V6–V8 regions by specific primers (without GC–clamp sequence). The PCR products were sequenced by Sanger method using the specific primers [25,26]. The DNA sequences were aligned in a reference database by BLAST from GenBank to find the closest known relatives.

The abundance of each taxonomic genus and species in all four groups of healthy children, acute diarrhea with unidentified pathogens, persistent diarrhea with the unidentified pathogens and virus-detected diarrhea was summarized in a histogram that was drawn by Microsoft Excel 2010 with the assistance of Krona—a complementary tool in Excel (http://krona.sourceforge.net, accessed on 19 April 2024). Based on the histogram, the diversity, distribution and abundance of each taxonomic rank were established.

### 2.7. Statistical Analysis

The data were analyzed by SPSS Statistics 20 software (IBM, Armonk, NY, USA). Differences were considered statistically significant when *p* ≤ 0.05.

## 3. Results

### 3.1. Extraction of Bacteria from Fecal Samples

**Figure 1:**
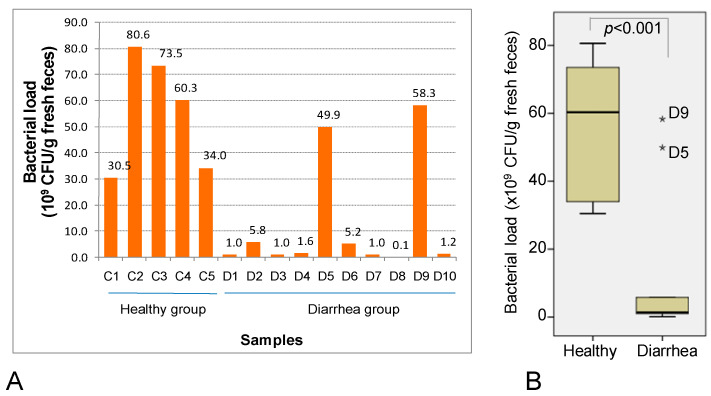
Descriptive graph (**A**) and statistical plot (**B**) of bacterial loads harvested from feces of healthy (C1–C5) and diarrheal children (D1–D10) aged 6–58 months old. *: The outliers.

The bacterial masses were harvested from the feces of 5 healthy and 10 diarrheal children from 6 to 58 months old. In general, bacterial mass from the healthy group was at a high permanence, from 3.1 × 10^10^ to 8.1 × 10^10^ CFU/g fresh feces (Figure 1A), with a mean of 5.6 × 10^10^ CFU/g and a median of 6.0 × 10^10^ CFU/g (Figure 1B). On the contrary, in the diarrheal groups, the bacterial cell masses were diverse, from a very low (0.1 × 10^9^ CFU/g fresh feces in sample D8) to high level (5.8 × 10^10^ CFU/g fresh feces in sample D9) (Figure 1A). Nevertheless, bacterial densities in the diarrheal feces presented a low downward tendency, 100-times lower when compared to the control group. Eight of the ten diarrheal samples contained extremely low bacterial loads, except samples D5 and D9, which harbored high bacterial density of 5 × 10^10^ and 5.8 × 10^10^ CFU/g fresh feces, respectively. After removing the two outliers from the data, the mean and median values of bacterial loads in the diarrheal group were 2.1 × 10^9^ CFU/g and 1.1 × 10^9^ CFU/g fresh feces, respectively (Figure 1B).

### 3.2. Selection of Appropriate Methods for Obtaining the Most Diverse Metagenomic DNA

**Figure 2:**
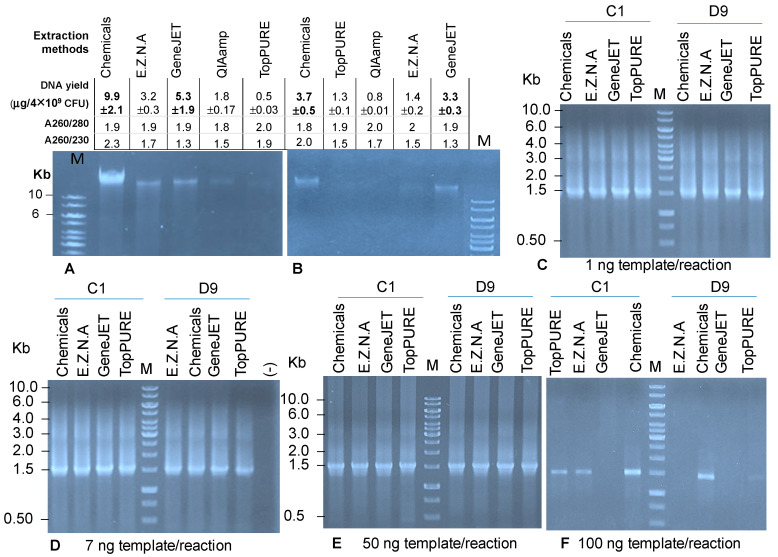
Nanodrop and electrophoresis analysis of the bacterial metagenomic DNAs extracted by five protocols and of metagene 16S rRNA amplified from difference concentrations of metagenomic DNA templates. M: 1 kb DNA marker (Fermentas); (**A**,**B**): metagenomic DNA extracted from healthy girl C1 and persistent diarrheal girl D9 respectively; (**C**–**F**): metagene 16S rRNA amplified from difference concentrations of metagenomic DNA templates at 1 ng, 7 ng, 50 ng and 100 ng per reaction, respectively; (-): negative control.

Bacterial cells from samples C1 and D9 were used for the extraction of metagenomic DNAs using the chemical method and four commercial kits. The obtained results (Figure 2A,B) indicated that the metagenomic DNA yields harvested from different protocols were different. The amounts of DNAs were higher in the samples extracted from a healthy child if compared to the ones extracted from a persistent diarrheal child. In both cases, the chemical method and kit GeneJET gave the highest yields (9.9 ± 2.1 μg from healthy child, 3.7 ± 0.5 μg from diarrheal child by biochemical method, and 5.3 ± 1.9 μg from healthy child, 3.3 ± 0.3 μg from diarrheal child by GeneJET kit, using the same bacteria load of 4 × 10^9^ CFU), followed by the E.Z.N.A. kit. The QIAamp and TopPURE kits produced lower DNA yields; however, QIAamp extracted the metagenomic DNA of bacteria from the stools of healthy children more efficiently than TopPURE did. On the agarose gel, metagenomic DNA extracted from the chemical method, GeneJET and E.Z.N.A kits was brightly observed at high molecular weight (>10 kDa) and was a little fragmented. The obtained results are in agreement with our previous study, in which both the chemical protocol and GeneJET kit gave the highest yields of bacterial metagenomic DNAs from the feces of children. However, in the previous study, the DNAs were extracted from the feces of a girl who suffered from intestinal infection with non-identified bacterial pathogens [19], while in this research, metagenomic DNAs were extracted from a girl who was diagnosed with persistent diarrhea with unidentified pathogens.

The electrophoresis technique has some advantages over ultraviolet absorbance for the qualification of DNA: (1) evaluation of the integrity of the extracted DNA; (2) estimation of the amount and length of target DNA fragments. However, the agarose electrophoresis cannot tell us the total mass of the DNA in a sample and the presence of contaminated proteins absorbed at A280 nm as well as aromatic compounds absorbed at A230 nm. The obtained results showed that, using the ultraviolet absorption method, all extracted metagenomic DNA samples were highly pure and free of protein contamination (geometric ≥ 1.8). However, some DNA samples possessed A260/230 lower than 1.7, which means the samples contained aromatic compounds, and these may negatively affect the polymerase activity in PCR or sequencing (Figure 2). In addition, the chemical protocol provided the highest ratio of A260/230 (geometric ≥ 2.0 in the samples from both healthy and diarrhea children). The DNA extracted by the GeneJET kit had the lowest A260/230 ratio (geometric = 1.3).

To check the presence of polymerase inhibitors in the extracted metagenomic DNAs, 16S rRNA PCR amplification was conducted in a total volume of 25 μL PCR reaction containing different DNA templates: 1 ng, 7 ng, 50 ng and 100 ng. As a result (Figure 2C–F), the metagene 16S rRNA of 1.5 kb was successfully amplified from both DNA templates derived from C1 and D9 in the case of DNA templates that increased from 1 ng to 50 ng/reaction (Figure 2C–E). However, at the highest template concentration (100 ng/reaction), the metagene was only amplified from the metagenomic DNAs extracted by the chemical protocol from both healthy and diseased girls or by TopPURE and E.Z.N.A kits from a healthy girl (Figure 2F). This suggested that the DNA extracted by GeneJET may contain more polymerase inhibitors than the ones extracted by the other four protocols. The DNA extracted from a diarrheal child was contaminated with higher inhibitors than the ones extracted from a healthy child. These results were consistent with the outcome of the qualitative evaluation using ultraviolet absorbance, in which DNA extracted by the chemical protocol possessed the highest ratio of A260/230 and the DNA extracted from the GeneJET kit presented the lowest ratio of A260/230. The E.Z.N.A and TopPURE kits provided impure DNA from a diarrheal girl. Taking all into account, the chemical protocol and the GeneJET kit stably reproduced the highest amount of bacterial metagenomic DNA with acceptable quality. The QIAamp, E.Z.N.A and TopPURE kits generated unstable amounts and quality of metagenomic DNAs.

**Figure 3:**
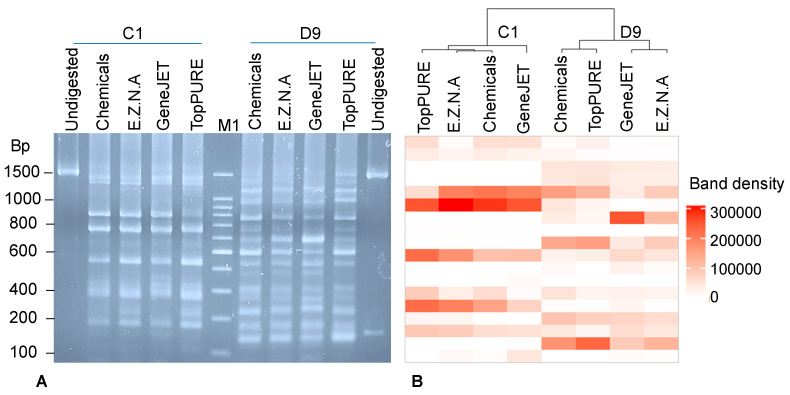
Electrophoresis analysis of restriction fragment-length polymorphism (RFLP) of the *Mbo*I-digested PCR products of the metagene 16S rRNA amplified from metagenomic DNA extracted samples C1, D9 (**A**) and cluster analysis of the RFLP profile (**B**). M1: 100 bp DNA marker (Intron, Gyeonggi-do, Republic of Korea).

In order to primarily find out the best kit for extracting diverse metagenomic DNAs of bacteria from the children’s stools, we digested the metagene 16S rRNA, which was amplified from metagenomic DNAs C1 and D9 extracted using four methods, with *Mbo*I. A total of 18 separated DNA fragments were visible on the 2.5% agarose gel (Figure 3A), while the PCR-RFLP profiles of the metagene 16S rRNAs derived from healthy and diarrheal children were different from each other, and the fingerprints of DNA bands originated from metagenomic DNAs extracted using four methods in each group were highly similar, with the only difference being the density of DNA bands in the healthy child (Figure 3A). The GeneJET kit generated bacterial metagenomic DNAs differing from the DNA extracted by the other kits. In the diarrheal child, the PCR-RFLP fingerprints created by DNAs extracted by GeneJET and E.Z.N.A kits were in a cluster (Figure 3B), while the fingerprints created by DNAs extracted by the chemical protocol and TopPURE kit were in a separated cluster. Considering all criteria, including quantity, quality and diversity, of the bacterial metagenomic DNAs, both the chemical method and GeneJET kit were chosen for the extraction of metagenomic DNA of bacteria from five control samples and ten diarrheal samples.

### 3.3. Bacterial Metagenomic DNA Extraction and Analysis of Bacterial Diversity in the Stool Samples Using Fingerprinting Techniques

**Figure 4:**
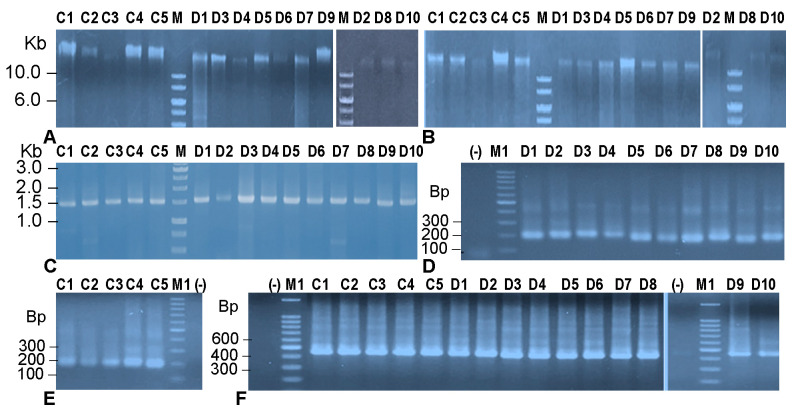
Analysis of metagenomic DNA extracted by chemical method (**A**), kit GeneJET (**B**), PCR products amplifying metagene 16S rRNA (**C**), fragment V3 (**D**,**E**) and fragment V6–V8 (**F**) of metagene 16S rRNA on 0.8% agarose gel. C1–C5: healthy samples, D1–D10: diarrhea samples; M: 1 kb DNA marker (Fermentas); M1: 100 bp DNA marker (Intron, Gyeonggi-do, Republic of Korea); (-): Negative PCR control, using water instead of DNA template.

**Figure 5:**
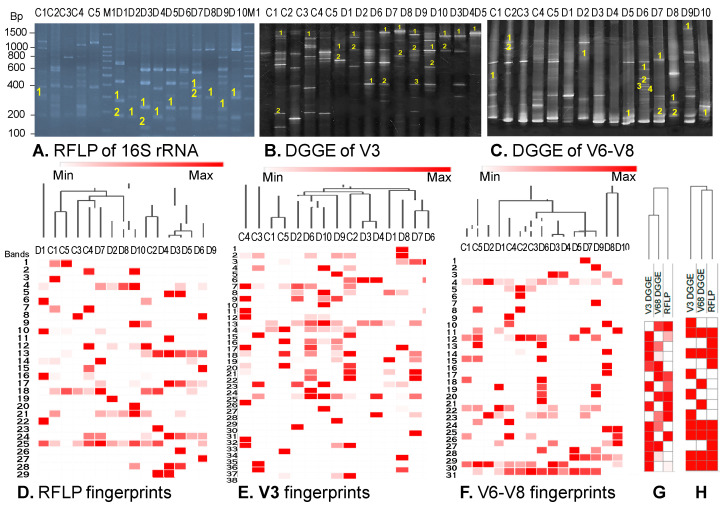
Analysis of metagene 16S rRNA (**A**), V3 region (**B**), V6–8 region (**C**) in metagene 16S rRNA of bacteria in fecal samples collected from healthy and diarrheal children by electrophoresis, and cluster analysis of fingerprints of RFLP (**D**) DGGE of V3 region (**E**), V6–V8 region (**F**); Cluster analysis of Shannon–Wiener diversity index (**G**) and Simpson’s diversity index (**H**) of the samples based on DGGE and RFLP profiles. C1–C5: Healthy samples, D1–D10: diarrheal samples; M1: 100 bp DNA marker (Intron, Gyeonggi-do, Republic of Korea). The numbers on the gel diagrams (**A**–**C**) indicate the order number and the site of DNA bands that were recovered for sequencing.

The metagenomic DNA of bacteria from fecal samples of five healthy and ten diarrheal children were extracted by chemical protocol and GeneJET kit. The results (Figure 4A,B) showed that metagenomic DNA masses from the same amounts of bacteria from diverse samples was different in both control and diarrheal groups and consistent between the chemical method and the GeneJET kit. The samples D2, D8, D10 gave the lowest yields of the metagenomic DNAs those correspond to the three youngest children in the disease group, with respect to 1, 6 and 8 months. That means DNA yield was depended on the samples’ natural properties. In addition, the metagene 16S rRNA (~1.5 kb) (Figure 4C) and the V3 (~200 bp) (Figure 4D,E), V6–V8 (~450 bp) (Figure 4F) regions of the 16S rRNA were successfully amplified from all samples. The metagene 16S rRNA of all samples was digested by *Mbo*I into a maximum of 29 different length fragments (Figure 5A). The V3 regions generated maximum 38 DNA bands (Figure 5B) and V6–V8 immigrated on the gel into a maximum of 31 DNA polyphormisms (Figure 5C). 

This result indicated that the DGGE profile of the V3 region had the highest diversity, and the RFLP profile had the lowest diversity. Statistical analysis based on the density and polymorphism of DNA bands showed that there was no significant difference between the control and disease groups. The DNA from healthy samples was not clustered or separated from diarrheal samples (Figure 5D–F). Cluster analysis of the Shannon–Wiener diversity index (Figure 5G) and Simpson diversity index (Figure 5H) of the samples based on DGGE and RFLP profiles indicated that fingerprints in the V3 region obtained by DGGE were the most diverse when compared to the other analysis.

**Figure 6:**
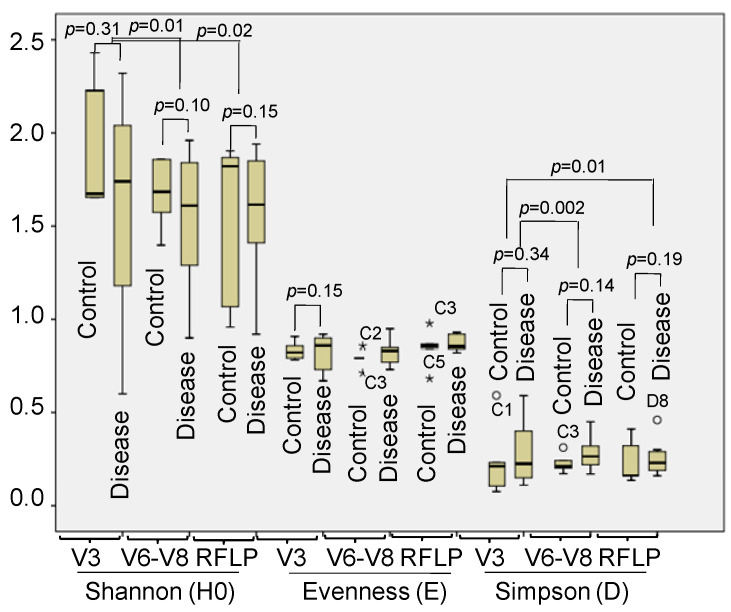
The differences in Shannon–Wiener diversity (H0) abundance or evenness (E) and Simpson’s diversity (D) indices were assessed through the profiles of DGGE of V3, V6–V8 regions and RFLP of metagene 16S rRNA digested with *Mbo*I. C1–C5: Healthy samples, D1–D10: diarrhea samples; *: outliers.

Based on statistical analysis of Shannon–Wiener diversity (H0), species abundance or evenness (E) and Simpson’s diversity (D), we found that the Shannon indices of V3 profiles exhibited a higher tendency than the profiles of V6–V8 or RFLP with significant differences (*p* = 0.01 and 0.02, respectively) (Figure 6). The Simpson and evenness indices of fingerprints of all V3, V6–V8 and RFLP were low. However, the Simpson indices of V3 profiles presented a significant difference from other profiles of DGGE analyzing V6–V8 and RFLP analyzing 16S rRNA (*p* = 0.02 and 0.01, respectively) (Figure 6). A higher Shannon index in V3 profiles expressed a higher diversity of bacteria. The above analysis did not show any significantly different indices between the control and disease groups, which may be due to the limited numbers of individuals in each group. However, a box plot suggested that the Shannon indices of the control group tended to be higher than the one in the disease group in both fingerprints in the V3 and V6–V8 regions. Thus, DGGE analysis of the V3 region is suitable for the assessment of bacterial diversity in the fecal samples in this study.

### 3.4. Sequencing the Representative DNA Bands of Individual Samples and Assessment of Bacterial Diversity

**Figure 7:**
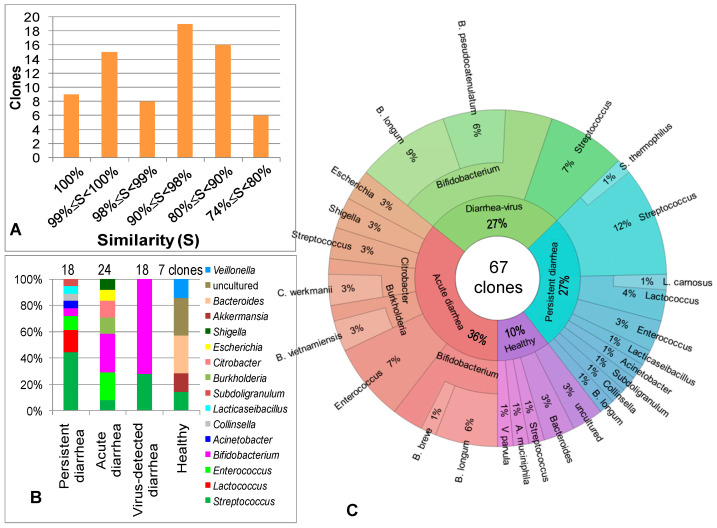
The similarity of 16S rRNA V3, V68 fragments’ sequences with the most similar genes from NCBI GenBank (**A**) and diversity of bacterial community groups identified by the sequences in healthy and diarrheal children at genus level (**B**) and species level (**C**).

A total of 73 clones of representative DNA bands (Figure 7A–C) were sequenced. The detailed sequences are provided in Supplementary Table S1, of which 9 sequences were 100% similar with corresponding genes from the NCBI GenBank; 67 sequences (accounting for 91.78%) were similar, over 80%, to the corresponding reference RNA sequences (refseq_rna) in GenBank (accessed on 3 May 2024) (Figure 7A, Table S1). Filtering out the sequences with similarity lower than 80%, then retaining genus names of the sequences possessing similarity in a range from 80 to lower than 99%, indicated that bacterial diversity was different, depending on the groups, such as healthy, acute diarrhea with unknown reason, virus-detected diarrhea and persistent diarrhea with unknown reason (Figure 7B). The diversity was independent with the RFLP or DGGE methods; hence, there was no difference between the two methods. The poorest bacterial diversity was observed in the feces of children infected with viruses. In contrast, the greatest bacterial diversity appeared in the feces of children suffering from unknown pathogenic persistent diarrhea (Figure 7C). The uncultured bacteria, *Bacteroides* genus and species *Akkermansia muciniphila* and *Veillonella parvula* were only seen in the healthy group, while *Bacteroides* was highly abundant in the healthy group but absent in the diarrheal groups. *Streptococcus* was increased in the group of viral diarrhea and reached the highest percentage in persistent diarrheal samples.

In the diarrheal groups, the most diverse bacteria were observed in the feces of children suffering from persistent diarrhea (8 genera/18 sequences), followed by acute diarrhea (7 genera/24 sequences). The poorest diversity of bacteria was found in the group of virus-detected diarrhea (2 genera/18 sequences) (Figure 7B,C). In virus-infected infants, 18 representative DNA bands from gels were sequenced. Among them, 13 sequences (accounting for 72%) were *Bifidobacterium,* and 5 sequences belonged to *Streptococcus* (28%). This means viral infections cause drastic changes in the intestinal microflora.

In acute diarrhea for unknown reasons, *Bifodobacterium* was the most dominant genus (accounting for 29%), followed by *Enterococcus* (accounting for 21%), *Burkhoderia* (13%), *Citrobacter* (13%), *Streptococcus* (8%), *Shigella* (8%) and *Escherichia* (8%). Except for two potential candidates of probiotics *Bifidobacterium* and *Enterococcus*, the remaining genera were potentially related to diarrhea. The bacteria detected in the acute diarrheal group were not on the list of bacteria that can be detected using the Allplex™ GI-Bacteria(I) kit (Seengene Inc., Seoul, Republic of Korea), except *Shigella*. *Bifidobacterium* and *Enterococcus* are supposed to be beneficial bacteria to self-rescue the microbial balance. However, the *Enterococcus* genus comprises over 50 species, of which many species relate to severe diseases with a prevalent multidrug resistance.

In the group of persistent diarrhea, *Streptococcus* emerged as the most dominant genus, accounting for 44%, followed by *Lactococcus* (17%), *Enterococcus* (11%), and equal percentages (6%) of *Lacticaseibacillus*, *Acinobacter, Collinsella* and *Subdoligranulum*. *Streptococcus* was found with a high ratio in the viral diarrhea group (accounting for 28%) but was also observed in the healthy group (holding 14%) and reduced in acute diarrhea (occupying 8%). The increase in *Streptococcus* is related to an increase in oxygen in the gut environment associated with persistent diarrhea without a sign of recovery from diarrhea. The increase in the *Streptococcus* population and reduction in *Bifidobacterium* indicated the dysbiosis of the gastrointestinal microbiota. The reduced tendency of the *Streptococcus* population is shown to be a sign of the recovery phase [28]. Therefore, patients suffering from acute diarrhea in this study may be in the disease recovery phase.

## 4. Discussion

### 4.1. The Separation of Bacteria from Fecal Residues Assists in Increasing the Quality of the Extracted Metagenomic DNAs; The Chemical Protocol and Kit GeneJET Are Good for the Extraction of the Efficient and Diverse Bacterial Metagenomic DNA

The human gastrointestinal tract contains a vast community of microbes that is of particular interest because of its high diversity and relevance to human health and disease. Using a culture method, a small percentage of these microbes were identified from fecal samples. Thus, to know the relationship of the bacterial community with certain diseases, nowadays, a metagenomic strategy based on next-generation sequencing has been increasingly exploited. However, fecal samples are very complex environments, comprising both biotic and abiotic compounds that affect the outcome quality. Hence, several procedures for the extraction of the quantitative and qualitative metagenomic DNA from feces have been investigated [17,18,29]. However, a common point of these studies is that they extracted DNAs directly from feces or did not fractionate the bacteria from remaining fecal residues, as performed in our study. Thus, the separation of bacteria from fecal compounds is a new point in this study with the aim to reduce polymerase inhibitors in the extracted DNA samples. However, to recover bacteria as completely as possible (including both free-living bacteria and fecal debris-impended bacteria), we diluted then homogenized the feces repeatedly combined with fractional centrifugation with a gradual increase in speed to remove crude residues first, then fine residues. The bacterial cells were washed to be ready for metagenomic DNA extraction. We found that bacterial loads in the diarrheal samples were much lower than the ones in the healthy control samples. This means that the diarrheal status reduces the richness of gastrointestinal bacteria. As supported by previous studies, gastrointestinal infections such as *Clostridium difficile* [30] and rotavirus [31] also led to a reduction in the bacterial load and diversity.

The metagenomic DNA of the separated bacteria from a healthy child (C1) and persistent diarrheal child (D9) was successfully extracted using the chemical method, as described by Sambrook et al. (2001), and four commercial kits, including TopPURE, E.Z.N.A, GeneJET and QIAamp. However, the chemical protocol and GeneJET kit gave the highest yields; on the contrary, QIAamp generated a low yield. In agreement with our obtained results, a previous investigation applied the phenol–chloroform technique (similar to the chemical protocol in this study), with or without a bead-beating step, a QIAamp Fast DNA Stool Mini Kit and a QIAamp Power Fecal Pro DNA Kit for the extraction of metagenomic DNA directly from stool samples of humans infected with parasites. The obtained results showed that the phenol–chloroform technique provided a four-times-higher DNA yield than the QIAamp kit did. However, the phenol–chloroform technique generated the lowest detection rate because of inhibitor contamination [17]. The mean ratios A260/280 and A260/230 of the metagenomic DNA extracted by the phenol–chloroform technique were 1.66 and 0.58 and, for the QIAamp kit, were 2.0 and 1.1, respectively [17]. Meanwhile, our results also indicated that the chemical protocol generated the highest DNA yield (four-times higher than QIAamp kit), whereas the extracted DNA still possessed good quality (the high ratio of A260/230 ≥2.0 in both DNAs from healthy and diarrheal children did not inhibit polymerase at the high concentration up to 100 ng/reaction). This means that the procedure to separate bacteria cells from fecal residues can aid in increasing the quality of the DNA extracted using the chemical method. The PCR inhibitors’ presence in stools encompasses complex polysaccharides, lipids, urate, salts, cellulose, etc. In diarrheal children, who were administrated with antibiotics, PCR inhibitors include many phenolic compounds, components of bacterial cells, bacterial toxins, etc. [15,17]. Thus, the centrifugation and washing to remove the residues which secreted in feces or attached to bacterial cell surfaces assisted facilitate the extraction of bacterial metagenomic DNA. To our best knowledge, for the first time in this study, the fragmentation of bacteria from fecal samples was conducted to improve the quality of the metagenomic DNA of bacteria in stool samples.

Normally, the QIAamp kit is evaluated to be a favorable kit for the extraction of metagenomic DNA from stool samples in many studies [18,29] using feces directly for extraction. The QIAamp Power Fecal Pro is thought to be better than the QIAamp DNA Stool Mini Kit for high quantitative and qualitative DNA extraction [17,32]. With the aim to establish a standard protocol for minimizing technical variation in metagenomic analysis, Costea et al. investigated 21 methods for extracting DNA from the same fecal samples at 21 collaborating laboratories in 11 countries and found that the QIAamp kit was reproducible and easy to automate, generating good-quality DNA [18]. However, the authors wondered whether this method was optimal for low-biomass samples. In the present study, the yields of metagenomic DNA extracted from samples D2, D8 and D10 were the lowest, which relates to the low-biomass stools (1 g of stool mass) harvested from very young children aged 1, 6 and 8 months, respectively. However, using the chemical protocol and GeneJET kit, we obtained metagenomic DNA for further analysis.

In healthy humans, the PCR inhibitors in the feces increase with age. In 2023, a study investigated four kits, including the QIAampPowerFecal Pro DNA kit (QPFPD, Qiagen, 40724 Hilden, Germany), MachereyNucleospin Soil (MNS, Macherey-Nagel, 67720 Hoerdt Cedex, France), MachereyNucleospin Tissue (MNT, Macherey-Nagel, 67720 Hoerdt Cedex, France) and MagnaPure LC DNA isolation kit III (MPLCD, Roche, 2720-413 Amadora, Portugal), for extracting metagenomic DNAs from both children and adult stools at low- and high-biomass samples, indicating that all the kits generated similar yields of DNA but were not sensitive enough for low-biomass samples [33]. On the other hand, the E.Z.N.A.^®^Stool DNA Kit (OMEGA Bio-tek Inc., Norcross, GA, USA) has been frequently used for the extraction of metagenomic DNA from stools. This kit was supposed to produce a comparable DNA yield to the QIAamp kit [17,18]. It is rare to investigate the GeneJET kit for extracting the DNA from feces. Thus, for the first time, this kit was successfully applied by our group for the extraction of the metagenomic DNA of bacteria from stool samples to obtain high yields with enough quality for polymerase activity. In addition, the analysis of PCR-RFLP showed that the metagene 16S rRNAs amplified from metagenomic DNA extracted using the chemical protocol and GeneJET kit were the most diverse. Thus, these methods were chosen for the extraction of the metagenomic DNA of bacteria from five control samples and ten diarrheal samples.

### 4.2. PCR-DGGE Fingerprints of V3 Region Are Suitable for Evaluation of Bacterial Diversity of Fecal Samples if Compared to the PCR-RFLP Fingerprints of 16S rRNA or PCR-DGGE Fingerprints of V6–V8 Region

The bacterial 16S rRNA is responsible for initiating protein synthesis in bacteria by recognizing and binding to the Shine–Dalgarno region in mRNA. The gene (over 1.55 kb) is divided into conserved domains and hypervariable domains that vary from bacteria. The hypervariable domains comprise nine hypervariable regions named V1 to V9, which alternate to the eight conserved regions of the conserved domains. Previous research reported that the V3–V5 region is suitable for identifying *Klebsiella*, and V1–V3 is good for the detection of *Escherichia* and *Shigella* [34], which are all related to diarrhea. The V3 is the best region for the analysis of bacterial diversity using the PCR–DGGE method [35], while the V6–V8 region is good for the analysis of bacteria belonging to diarrhea-related bacteria such as *Clostridium* and *Staphylococcus* genera [36]. The DGGE separates the same-length PCR products into different bands on polyacrylamide gel based on their nucleotide sequences specified by Tm. The DGGE has been successfully used for the identification of diverse bacterial contamination, including *B. cereus*, *B. licheniformis*, *Enterobacter cloacae*, *Acinetobacter* spp., *Xanthomonas/Pseudomonas* spp. *Staphylococcus/Lysinibacillus*, *Enterobacter* spp., *S. epidermidis*, *Klebsiella* spp. and *Serratia* spp., in commercial probiotic formulations, while culturing techniques were not able to detect these bacteria [37]. The DGGE is also commonly used to investigate the relationship between bacterial communities in feces with certain diseases, such as allergies (using V6–V8 or V3 regions of 16S rRNA) [38,39] and eczema (using V2–V3 region) [40]. By using the V2–V3 region, PCR–DGGE has also been applied for the assessment of dysbiosis in inflammatory bowel disease [41]. The PCR–DGGE analysis of the V6–V8 region was applied to identify the diversity and etiology of human diarrhea and indicated that bacterial community and diversity were reduced in the diarrheal feces. However, the DGGE could not separate the V6–V8 region of *E. coli* and *Shigella* spp. [42]. In this study, the analysis of the V3 region in the metagene 16S rRNA of bacteria in diarrheal children’s feces using the DGGE method also gave more diverse results rather than the V6–V8 region or by using the RFLP method analysis of the 16S rRNA metagene, which was presented by a higher Shannon H0 diversity index and lower Simpson index, with the significant differences observed through statistical analysis (*p* < 0.05). However, this is the first study investigating the DGGE method for the analysis of the V3 and V6–V8 regions and RFLP for the analysis of metagene 16S rRNA to assess bacterial diversity in the feces of diarrheal children.

### 4.3. Viral Diarrhea Has the Strongest Impacts on the Bacterial Diversity; Streptococcus May Be an Indicator of Persistent Diarrhea, and Probiotic Strains May Relate to the Self-Recovery of the Diarrhea

To identify some dominant bacteria in the fecal samples, a total of 49 representative DNA bands on RFLP and DGGE gels were sliced out for sequencing to obtain 73 separated sequences, whereas 91.78% of the sequences possessed similarity over 80% with the corresponding reference RNA sequences (refseq_rna) in the NCBI GenBank (accessed in 3 May 2024). By filtering out the sequences with low identity (<80%), we found 16 bacterial genera present in the stools. In terms of bacterial diversity, the bacterial communities in the persistent diarrheal feces were the most diverse, with a Shannon index of 1.7 corresponding to 8 genera/18 sequences. The diversities of bacteria in the stools of acute diarrhea and healthy children were similar with H0 = 1.6. Notably, the lowest Shannon diversity index (H0 = 0.6) was seen in the bacterial communities of virus-infected infants corresponding to the 2 genera/18 sequences and was significantly different from the other groups. Thus, the viruses impacted strongly on the gastrointestinal bacteria. In terms of the genera diversity, we only found two genera in this group, while *Bifidobacterium* accounted for 72%, and *Streptococcus* possessed 28%. Consistent with this result, the bacterial diversity in the feces of children with viral diarrhea was observed as being significantly reduced, while *Streptococcus* increased [31]. *Streptococcus* spp. and *Enterococcus* spp. are also reported to have a greater abundance in rotavirus-infected acute gastroenteritis infants, with a mean age of 11.8 months, and norovirus-infected acute gastroenteritis infants, with a mean age of 8.8 months, if compared to the control group. However, *Lactobacillus and Bifidobacterium* spp. are more predominant in rotavirus-infected children [43]. The increased abundance of intestinal probiotics such as *Lactobacillus* and *Bifidobacterium* may serve in the self-regulation and recovery abilities of the intestinal microbiota.

Although Shannon indices of the bacterial community in the feces of acute diarrheal and healthy children were similar, the bacteria detected in these groups were different. In terms of beneficial bacteria, *Bifidobacterium* and *Enterococcus* were the most dominant genera in the acute diarrheal group (accounting for 29% and 21% of the detected bacteria, respectively), while uncultured bacteria, *Bacteroides* genus and species *Akkermansia muciniphila* and *Veillonella parvula* were only seen in the healthy group (Supplementary Table S1, Figure 6) and not observed in the diarrheal groups. *Bifidobacterium* and *Enterococcus* are supposed to be beneficial bacteria to self-rescue the microbial balance. *Enterococcus* was the most dominant in the feces of acute diarrhea with unidentified pathogenic agents in this study, which was supposed to possess the ability to adsorb norovirus on the bacteria to inhibit them from dissemination [44]. *E. faecium* SF68 was evaluated to be an effective and safe probiotic for the treatment of acute diarrhea, even with antibiotic-resistant diarrhea [45]. However, the *Enterococcus* genus comprises over 50 species, and many species relate to severe diseases with a prevalent multidrug resistance. They are found at abundant levels in diarrheal individuals infected with different gastroenteritis pathogens [28]. *Bacteroides* is more abundant in healthy infants and then reduces with age; however, *Streptococcus* possesses a steady increase after the first year of life [46,47,48]. *Bacteroides* was not found in diarrheal children, but *Streptococcus* was increased in the group of viral diarrhea and reached the highest percentage in persistent diarrhea. In agreement with this study, the reduced proportion of *Bacteroides* in feces was also found in vitamin-D-deficient or obese children [46,48,49]. *Akkermansia muciniphila* is a mucin-degrading bacterium that is abundant in the feces of healthy children, potentially improving children’s growth [50], and great promise for cancer or metabolic disorder treatment [51]. The low abundance of this species in feces usually relates to children with allergic asthma [52] and autism [53]. Alongside beneficial bacteria, in the acute diarrhea group, some genera that may relate to the disease were detected, such as *Burkhoderia*, *Citrobacter*, *Streptococcus*, *Shigella* and *Escherichia*.

The bacterial community in the persistent diarrheal feces was the most diverse in this study, defined by an increase in the *Streptococcus* population (accounting for 44% of the community). This genus was found to have a high ratio (accounting for 28%) in the viral diarrhea group and was also observed in the healthy group (holding 14%) and reduced in acute diarrhea with unknown reasons (occupying 8%). The increase in *Streptococcus* is related to an increase in oxygen in the gut environment associated with persistent diarrhea without a sign of recovery of diarrhea. Thus, this genus also had the highest abundance in the persistent diarrhea group when compared to other diarrhea, including virus-infected and acute diarrhea. In this group, the *Bifidobacterium* population was reduced. The depletion of *Bifidobacterium* in this group is also a sign of non-viral diarrhea. In fact, the individuals in this group were negative for human Adenovirus F40/F41, Norovirus GI, Norovirus GII, Rotavirus A, Astrovirus, Sapovirus GI, GII, GIV, GV by QIAstat-Dx Gastrointestinal Panel (Qiagen). However, the reduction in *Bifidobacterium* or the increase in *Streptococcus* only reveals the dysbiosis of the microbiota; however, we did not find any specific pathogen responsible for the persistent diarrhea situation. Investigating the gut microbiota in 42 children diagnosed with autism spectrum disorder, Wang et al. indicated that the higher abundance of both *Streptococcus* and *Lactococcus* is a signature of the chronic gastrointestinal problem of children with autism [54]. *Lactococcus* comprises 12 species; however, this genus does not cause serious disease in humans, except *L. garvieae*, which can cause gastrointestinal disorders [55] before engendering other clinical presentations in 75% infective humans, such as infective endocarditis, liver abscess, septicemia with multiorgan failure, diverticulitis, etc. [56]. In addition to this, *L. lactis* has been reported to cause serious infections, such as endocarditis, peritonitis, and intra-abdominal infections in humans [57]. *Acinetobacter* comprises 38 species and is usually isolated from nosocomial infections. Some species of this genus admittedly cause serious public health problems, especially emphasizing their resistance to carbapenems and colistin, and may transfer resistance genes to other bacteria [58]. Some studies have indicated an association between *Acinobacter* with acute gastroenteritis [59,60], and there is evidence that gastrointestinal symptoms are in the early phase during development to enterogenic sepsis by *A. baunannii* [61]. Conversely *Acinetobacter* and *Collinsella* were usually found in patients with symptomatic atherosclerosis and rheumatoid arthritis. There is no evidence for an association between human diarrhea with *Collinsella,* but this genus was reported to increase populations in the feces of chronic diarrhea in monkeys [62].

Taking all this into account, although the number of samples was limited, with the diverse types of diarrhea in children, this research revealed the profits of bacterial extraction from fecal samples before extracting metagenomic DNAs for molecular analysis. The bacterial extraction increases the quality of metagenomic DNA derived from gastrointestinal bacteria. The chemical protocol and GeneJET kit proved capable of giving a high yield of metagenomic DNA from bacteria in low-mass fecal samples. Based on PCR fingerprinting analysis, dysbiosis of dominant bacteria was observed, and we preliminarily noted that the dysbiosis was typical for the type of diarrhea (viral infection or acute diarrhea or persistent diarrhea with unidentified pathogens). However, with limited samples in each group of diarrheal types, the bacterial dysbiosis tendencies in the groups were not affirmed. On the other hand, the results of the PCR-based fingerprinting techniques were partly dependent on the specificity and universality of primers. Hence, the bacterial diversity in the samples will be evaluated through metagenomic sequencing in our future research.

## 5. Conclusions

For the first time, the chemical protocol and GeneJET kit were successfully adopted to efficiently extract metagenomic DNAs with high quality and diversity, with the assistance of the bacterial separation process conducted from fecal samples. By sequencing the representative DNA bands from DGGE and RFLP gels, the dysbiosis of bacteria in gastrointestinal tracts was detected in persistent diarrheal children with an increase in the *Streptococcus* population and depletion of the *Bifidobacterium* population; viral infection strongly impacted the bacterial diversity in the feces with the sign of increasing *Bifidobacterium*. Beneficial bacteria were dominant in the healthy children.

## Figures and Tables

**Table 1:** The list of fecal samples harvested in Vietnam National Children’s Hospital used for this research.

Samples	Ages in Months	Gender	Health Condition	Diagnostic Result	Test Kit
C1	8	Female	Healthy	Negative	QIAstat-Dx^®^ Gastrointestinal Panel (Qiagen, Hilden, Germany)
C2	7	Female	Healthy	Negative	QIAstat-Dx^®^ Gastrointestinal Panel (Qiagen, Hilden, Germany)
C3	6	Male	Healthy	Negative	QIAstat-Dx^®^ Gastrointestinal Panel (Qiagen, Hilden, Germany)
C4	8	Male	Healthy	Negative	QIAstat-Dx^®^ Gastrointestinal Panel (Qiagen, Hilden, Germany)
C5	14	Female	Healthy	Negative	QIAstat-Dx^®^ Gastrointestinal Panel (Qiagen, Hilden, Germany)
D1	14	Female	Acute diarrhea	Negative	Allplex™ GI-Bacteria(I) Assay (Seegene Inc., Seoul, Republic of Korea)
D2	1	Male	Acute diarrhea	Negative	Allplex™ GI-Bacteria(I,II) Assay (Seegene Inc., Seoul, Republic of Korea)
D3	24	Female	Acute diarrhea	Adenovirus	Allplex™ GI-Virus Assay (Seegene Inc., Seoul, Republic of Korea)
D4	16	Male	Acute diarrhea	Adenovirus	Allplex™ GI-Virus Assay (Seegene Inc., Seoul, Republic of Korea)
D5	11	Male	Acute diarrhea	Enterovirus	Allplex™ respiratory panel Assay (Seegene Inc., Seoul, Republic of Korea)
D6	25	Male	Acute diarrhea	Negative	Allplex™ GI-Bacteria(I,II) Assay (Seegene Inc., Seoul, Republic of Korea)
D7	58	Female	Acute diarrhea	Negative	Allplex™ GI-Bacteria(I) Assay (Seegene Inc., Seoul, Republic of Korea)
D8	8	Male	Persistent diarrhea	Negative	QIAstat-Dx^®^ Gastrointestinal Panel (Qiagen, Hilden, Germany)
D9	11	Female	Persistent diarrhea	Negative	QIAstat-Dx^®^ Gastrointestinal Panel (Qiagen, Hilden, Germany)
D10	6	Male	Persistent diarrhea	Negative	QIAstat-Dx^®^ Gastrointestinal Panel (Qiagen, Hilden, Germany)

## Data Availability

The data are available in the Supplementary Materials.

## Supplementary Materials

The following supporting information can be downloaded at: https://www.mdpi.com/article/10.3390/idr16050075/s1, Table S1: Detailed sequences and analysis of the sequences of 73 clones derived from the DNA fragments, which was representative DNA of individual samples, extracted from PCR-RFLP and PCR-DGGE gels.
